# Presence of children in the home and intimate partner violence among women seeking elective pregnancy termination

**DOI:** 10.1371/journal.pone.0186389

**Published:** 2017-10-12

**Authors:** Corinne Peek-Asa, Audrey F. Saftlas, Anne B. Wallis, Karisa Harland, Penny Dickey

**Affiliations:** 1 Department of Occupational and Environmental Health, College of Public Health, University of Iowa, Iowa City, IA, United States of America; 2 Department of Epidemiology, College of Public Health, University of Iowa, Iowa City, IA, United States of America; 3 Department of Epidemiology and Population Health, School of Public Health & Information Sciences, University of Louisville, Louisville, KY, United States of America; 4 Department of Emergency Medicine, University of Iowa, Iowa City, IA, United States of America; 5 Planned Parenthood of the Heartland, Des Moines, IA, United States of America; University of Westminster, UNITED KINGDOM

## Abstract

**Introduction:**

Growing evidence identifies adverse health effects for children who witness intimate partner violence at home. Research has also identified that women seeking elective pregnancy termination are at high risk for partner violence. However, little is known about the risk for violence exposure among the children of women seeking elective pregnancy termination.

**Methods:**

We conducted a cross-sectional study of 957 women seeking elective pregnancy termination at a large family planning clinic. All subjects completed a 10-minute, anonymous questionnaire administered by computer in a private room. Our main outcome was 12-month prevalence of physical and/or sexual violence by a current or former partner using the Abuse Assessment Screen instrument. The presence of children under the age of 18 living with the respondent was the main exposure variable.

**Results:**

Women with children in the home had more than twice the odds of reporting physical and/or sexual IPV in the past year than women with no children, controlling for age (AOR: 2.23; 95% CI: 1.41–3.85). The increased odds of IPV among women with children as compared to women with no children was present across nearly all sociodemographic and lifestyle characteristics, and significantly higher for the youngest women (18–20 years). The highest odds for abuse occurred among women with children living at home, in a current relationship but not living with their current partner, and abused by a former partner (AOR = 10.9; 95% CI: 3.07–38.4).

**Conclusion:**

Nearly one of every 14 children identified in this study lived in a home with IPV. These findings support the development of IPV interventions that are family-centered, as well as the integration of trauma-informed care into healthcare settings. Healthcare visits for contraception and pregnancy termination may be ideal opportunities for implementation of screening and family violence interventions.

## Introduction

In 2006, the World Health Organization estimated the global lifetime prevalence of partner violence against women at 30% [1]. United States population-based estimates indicate that between 4 and 6 million women are physically or sexually assaulted annually by either a current or former intimate partner [2].

Effects of intimate partner violence (IPV) on women include serious injury and death as well as a wide range of physical and mental health outcomes, including sexually transmitted disease, chronic pelvic and abdominal pain, sexual dysfunction, substance abuse, suicide attempts, depression, and post-traumatic stress disorder [3–5]. Some studies report that abused pregnant women are at increased risk of adverse pregnancy outcomes such as miscarriage, premature birth, and low infant birth weight [2,6].

A plethora of negative health outcomes are associated with IPV against pregnant women. Amidst the many physical and psychological complexities include concern for the future safety of the unborn child and of the safety of the mother post-birth, which may lead a victim to consider elective pregnancy termination. A recent meta-analysis indicated that nearly a quarter of women seeking elective pregnancy termination were at one time victims of intimate partner violence [7]. Elective pregnancy termination among women with IPV was associated with sexual assault, lack of control over contraceptive choices, and coercive decision-making, and women who had not informed their partner about the termination were 3 times more likely to be victims of IPV than women who disclosed to their partner [7]. Studies that have examined factors influencing a woman’s decision to terminate a pregnancy find that relationship problems and concern for the effects on existing children are common themes [8]. Furthermore, this research shows that most women’s decisions involve complex and multifactorial issues, indicating that the co-occurrence of these concerns is common.

The National Survey of Children’s Health conducted in 2012 by the National Center for Health Statistics estimated that 7% of US children witness domestic violence in the home each year [9]. A growing body of research ties adverse child experiences, including witnessing violence in the home, to lifelong adverse health consequences [10]. Children exposed to IPV in the home are at risk for behavioral and emotional problems, poor academic performance, and adverse health outcomes [11–13].

Most studies of IPV among women seeking pregnancy termination have relatively small sample sizes, and none have examined the prevalence of current children in the home. The objective of this study, conducted in a large clinical population of women seeking pregnancy termination, was to examine the prevalence of children living in the home in relation to reporting of intimate partner violence among women seeking elective abortions.

## Methods

### Study setting and participants

We conducted a cross-sectional, computer-based, self-administered, anonymous survey to determine the prevalence of intimate partner violence among women seeking elective pregnancy termination. The study population consisted of all pregnancy termination clients seen at a large Midwestern family-planning clinic that provided surgical (aspiration) and medical termination over the 8-½ month study period of November 1, 2007 through July 18, 2008.

### Study sample and protocol

Eligible subjects were 18 years or older, state residents, and had reading proficiency in English or Spanish. Women returning for re-aspiration procedures were not eligible. Following clinic intake, staff introduced the study to eligible patients in a private room. All participants provided informed, voluntary consent and completed the computer-based questionnaire (English or Spanish) in a private room with the door closed.

Following clinic intake and prior to any procedures or administration of any medication, health educators introduced the study to eligible patients in a private clinic room. Each woman read a brief IRB-approved description of the study containing the elements of voluntary consent, printed on a laminated sheet. If interested, participants reviewed the written consent form, signed it, and then completed the 10-minute questionnaire in a private room. Participants were informed that their responses were anonymous, and that their providers would not see their responses. In order to address any safety or security issues, all women in the clinic were provided with resources about intimate partner violence. Each participant was informed on the survey that they could ask to speak with a social worker. Since the survey was anonymous, no specific response follow-up was conducted. At completion, each participant received a $5.00 gift card as compensation for her time. Eligibility, invitation to participate, signed consent, and questionnaire completion was recorded by clinic staff.

The study was approved by the University of Iowa Institutional Review Board and the clinic Institutional Review Board. Before implementing the study, all clinic staff attended a three-hour training session conducted by the Children and Families of Iowa Family Violence Center. The training session covered the dynamics of IPV and how it impacts victims and their families, safety planning, victim advocacy, and available state and local support services.

### Measures of IPV

Sociodemographic items included participant’s age, race/ethnicity, education, employment, health insurance, relationship status, and presence and ages of children under 18 living in the participant’s household. A modified version of the Abuse Assessment Screen (AAS) was used to assess physical and sexual abuse during the prior year [14]. Specifically, subjects were asked: “*Within the last year*, *have you been hit*, *slapped*, *kicked*, *or otherwise physically hurt by anyone?*” and “*Within the last year*, *were you forced to have sex or engage in a sex act when you didn't want to?* Perpetrators were identified as a “current partner”, “ex-partner”, family member(s), person(s) known to the subject, stranger(s), or as “someone else.”

Alcohol use was evaluated using quantity-frequency items adapted from the National Institute on Alcohol Abuse and Alcoholism [15]. Women reported alcohol consumption and drug use, including marijuana/hashish, cocaine/crack, amphetamines or methamphetamines, etc., based on the 3 months before they found out they were pregnant. Alcohol consumption was calculated as the number of drinks per week (i.e., number of drinking days per week multiplied by the average number of drinks per day). We categorized alcohol consumption for women and men based on the corresponding frequency distributions in the study population. Per the NIAAA guidelines, binge drinking for women was defined as consumption of 4 or more drinks during a 2-hour period; and 5 or more drinks in a 2-hour period for men.

Depressive symptoms were measured using a modified version of the 10-item Center for Epidemiologic Studies Depression Scale [16], which was modified by asking women about symptoms experienced in the prior month, rather than the prior week. To analyze depressive symptomology, we calculated total CES-D scores and created three categories (low = <10, moderate = 10–14, and high ≥15) of depressive symptomology based on the distribution of the data. This method differs from the recommended CES-D scoring because we altered the temporality of the measure.

We created four categories for participant age in years (18–20, 21–24, 25–29, 30–44); these categories were collapsed into three (18–20, 21–24, and 25+) for multivariate analyses. Race/ethnicity was categorized as white, black, Latina, and non-Latina. Due to low numbers of non-white participants (reflective of Iowa’s population), we categorized data for multivariate analyses as white/non-white. Employment was dichotomized as employed/not employed. Additional variables included health insurance (private and public, with public insurance and no insurance collapsed into one category), medical or surgical termination, partner status (current, former, unknown), presence/absence of children <18 years in the household (dichotomous variable), number of children (1, 2, and 3+), and mean child age (in years) among participants with children.

### Data analysis

The study participation rate was calculated as the number of women who completed the questionnaire divided by the total number eligible; the response rate was calculated as the number of women who completed the questionnaire divided by the number invited to participate. Demographic data were assessed using descriptive statistics to evaluate measures of central tendency, frequency, and percent.

We calculated prevalence rates of physical and sexual abuse reported within the past 12 months for all women. For partner-specific rates (current, former, etc.), prevalence was calculated as the number of women who met category-specific abuse criteria divided by the total number who responded to the relevant category-specific questions. Chi-square tests were used to evaluate significant differences in prevalence rates by sociodemographic and lifestyle variables.

To describe the association of the presence of children with IPV we calculated age-adjusted odds ratios (ORs) and 95% confidence intervals (CIs). We examined this association across strata of covariates in order to compare odds among study subgroups. We used multivariable logistic regression procedures to evaluate the relationships and effect modification among physically and/or sexually abused women with children living in their household, perpetrator, and partner status.

## Results

Of 1,415 pregnancy termination clients seen in the clinic over the study period: 1,193 were eligible, 1,108 were invited to participate, 990 consented, and 986 completed the questionnaire. Participation was high: 82.6% (986/1,193) of women eligible for the study filled out the survey. For this analysis, we examined data from the 957 participants with non-missing responses to physical and sexual abuse items and number of children in the home.

Characteristics of the overall sample and prevalence rates have been reported previously [17]. To summarize, 9.9% reported physical abuse and 2.3% sexual abuse by a current or former partner. The combined prevalence of *any* physical and/or sexual abuse perpetrated by a current or former partner was 10.8%. The 26% of respondents not in a current intimate relationship reported the highest prevalence of physical and/or sexual IPV (16.0%), which by definition was perpetrated by a former partner.

Of the 957 participants included in this analysis, 603 (63.0%) reported that they lived with children under the age of 18. Of those 603, 491 (81.4%) households had either one or two children, and an additional 112 (18.6%) lived with three or more children. Of the 1,064 children identified, almost half were under the age of 4 (512; 48.1%), with a mean age of 5.8 years (SD: 4.5).

Having children was associated with being older, being more likely to be non-white, and having less educated than women reporting no children (Table 1). Almost three-quarters of women with no children (262; 74.0%) reported either full- or part-time employment as compared to 69.3% (418) of women with children. Not having children was associated with being on public insurance or having no insurance coverage. Child status was not significantly associated with relationship status; however, women with children were significantly more likely to report cohabitating with a partner (233; 70.4%) as compared to women without children (98; 29.6%).

**Table 1:** Demographic characteristics of participants seeking induced abortion at a women’s health clinic, by presence of children in the household, November 2007-July 2008. (N = 957).

Participant Characteristics	Participants: With Children in Home (N = 603) N (%)	Participants: No Children in Home (N = 354) N (%)	p-value
**Number of children in household (mean/SD)**	1.8 (1.0)	---	---
1	284 (47.1)	---	---
2	207 (34.3)	---	---
3+	112 (18.6)	---	---
**Ages of children in household (mean/SD)**	5.8 (4.5)	---	---
<1 year	90 (8.4)	---	---
1–4 years	422 (39.7)	---	---
5–9 years	315 (29.6)	---	---
10+ years	237 (22.3)	---	---
**Participant age (Mean/SD)**	27.4(6.1)	22.7(4.3)	< .01
18–20	74(12.3)	126(35.6)	
21–24	146(24.2)	136(38.4)	
25–29	177(29.3)	68(19.2)	
30–44	202(33.5)	23(6.5)	
Missing	4(0.7)	1(0.3)	
**Participant race**			
White	479(79.4)	311(87.8)	< .01
Black	77(12.8)	24(6.8)	
Other	39(6.5)	19(5.4)	
Missing	8(1.3)	0	
**Participant ethnicity**			
Latina	51(8.5)	25(7.1)	0.41
Non-Latina	544(90.2)	329(92.9)	
Missing	8(1.3)	0	
**Participant education**			
≤ High school or less	213(35.3)	96(27.1)	< .01
> High school	390(64.7)	258(72.9)	
**Participant employment status**			
Full-time	312(51.7)	167(47.2)	< .01
Part-time	106(17.6)	95(26.8)	
No employment	173(28.7)	85(24.0)	
Missing	12(2.0)	7(2.0)	
**Participant insurance status**			
Private Insurance	240(39.8)	161(45.5)	< .01
Public Insurance	159(26.4)	26(7.3)	
No Insurance	186(30.9)	133(37.6)	
Missing	18(3.0)	34(9.6)	
**Participant relationship status**			
Current partner	432 (71.6)	268 (75.7)	0.18
No current partner	158 (26.2)	83 (23.5)	
Unknown partner status	13 (2.2)	3 (0.8)	
**Participant cohabitation status (only known for participants reporting a current partner; n = 700)**			
Yes	233 (70.4)	98 (29.6)	< .01
No	189 (27.0)	167 (62.3)	
No current partner/unknown partner status	171 (28.4)	86 (24.3)	
Missing	10 (1.4)	3 (0.4)	
**Abortion type**			
Medical	279(46.3)	186(52.5)	0.07
Surgical	313(51.9)	163(46.1)	
Missing	11(1.8)	5(1.4)	
**Depressive symptoms (CES-D)**			
Low (<10)	294(48.8)	195(55.1)	0.16
Medium (10–14)	135(22.4)	78(22.0)	
High (≥15)	152(25.2)	73(20.6)	
Missing	22(3.6)	8(2.3)	
CES-D score (Mean/SD)	9.9(6.6)	9.4(6.4)	0.25
**Participant alcohol use per week**			
None	167(27.7)	56(15.8)	< .01
0.1–4 drinks	265(43.9)	141(39.9)	
4.1–14 drinks	75(12.4)	78(22.0)	
>14 drinks	42(7.0)	52(14.7)	
Missing	54(9.0)	27(7.6)	
**Participant binge drinking (≥4 drinks in 2 hours)**			
No alcohol use	167(27.7)	56(15.8)	< .01
Alcohol use/no binge drinking	176(29.2)	99(28.0)	
≤ 1/month	130(21.6)	86(24.3)	
> 1/month	102(16.9)	98(27.7)	
Missing	28(4.6)	15(4.2)	

Overall, having children was associated with drinking less alcohol, with just over one-quarter of women with children (27.7% vs. 15.8% for women with no children) reporting no alcohol use (Table 1). Not having children was associated with consuming a greater quantity of drinks per week and significantly higher proportion of binge drinking (i.e., consumption of ≥4 drinks in a two-hour period) per month (27.7% for women with no children vs. 16.9% for women with children).

After adjusting for participant age (Table 2), the prevalence of physical and/or sexual IPV for women with children living at home was more than double that of women with no children in their household (AOR: 2.33; 1.41–3.85). We examined the odds of physical and sexual abuse for women with children living at home compared to those without for a variety of characteristics. The odds of abuse for women ages 18–20 years old with children was 4.69 times higher (95% CI 1.83–12.03) than for women without children; the older age groups did not show a significantly increased odds for abuse by child status. Women with the following characteristics had a significantly higher odds of abuse if they had children living at home than women who did not: white race (AOR 2.18; 1.27–3.74), non-Latina ethnicity (AOR: 2.76; 1.62–4.68), at least a high school diploma (AOR 2.62; 1.32–5.18), unemployed (AOR 3.48; 1.24–9.66), private health insurance (AOR 2.85; 1.14–7.16), have a current partner (3.09; 1.59–6.03); high levels of depressive symptomology (AOR 2.72; 1.13–6.55); had a medical termination (AOR 3.21; 1.44–7.18); light (AOR 3.08; 1.45–6.53) moderate (AOR 4.02; 1.35–11.97); and non-binge drinking (AOR 4.15; 1.73–9.96).

**Table 2:** Prevalence rate and odds ratios (crude and age-adjusted) of physical/sexual IPV associated with having children <18 years in the household among participants seeking induced abortion at a women’s health clinic, November 2007-July 2008.

	Physical IPV (n = 96)		Sexual IPV (n = 24)		Physical or sexual IPV (n = 106)			
	Prev. rate per 100 (n)^a^		Prev. rate per 100 (n)^a^		Prev. rate per 100 (n)^a^		OR (95% CI) ^R^	
	Kids	No kids	Kids	No Kids	Kids	No Kids	Crude	Age-Adjusted
**All women**	12.3(74)	6.2(22)	2.8(17)	2.0(7)	13.4(81)	7.1(25)	2.04(1.28–3.27)	2.33(1.41–3.85)
**Age**								
18–20	20.3(15)	4.8(6)	4.1(3)	0.8(1)	21.6(16)	5.6(7)	4.69(1.83–12.03)	
21–24	8.9(13)	8.1(11)	5.5(8)	2.2(3)	13.0(19)	9.6(13)	1.42(0.67–2.99)	
25+	11.9(45)	5.5(5)	1.3(5)	3.3(3)	11.9(45)	5.5(5)	2.32(0.89–6.02)	
**Race**								
White	11.9(57)	6.4(20)	2.7(13)	2.3(7)	12.9(62)	7.4(23)	1.86(1.13–3.07)	2.18(1.27–3.74)
Non-white	13.8(16)	4.7(2)	3.5(4)	0	15.5(18)	4.7(2)	3.77(0.84–16.97)	4.43(0.90–21.84)
**Ethnicity**								
Latina	5.9(3)	12.0(3)	3.9(2)	4.0(1)	5.9(3)	12.0(3)	0.46(0.09–2.45)	0.29(0.04–2.05)
Non-Latina	12.9(70)	5.8(19)	2.8(15)	1.8(6)	14.2(77)	6.7(22)	2.30(1.40–3.78)	2.76(1.62–4.68)
**Education**								
≤ High school	15.0(32)	11.5(11)	2.8(6)	1.0(1)	16.9(36)	11.5(11)	1.57(0.76–3.24)	1.65(0.77–3.53)
> High school	10.8(42)	4.3(11)	2.8(11)	2.3(6)	11.5(45)	5.4(14)	2.27(1.22–4.23)	2.62(1.32–5.18)
**Employment**								
Employed	10.7(45)	6.5(17)	2.1(9)	2.3(6)	11.9(50)	7.6(20)	1.63(0.95–2.80)	1.92(1.07–3.46)
Not employed	15.6(27)	5.9(5)	4.7(8)	1.2(1)	16.8(29)	5.9(5)	3.22(1.20–8.65)	3.46(1.24–9.66)
**Health insurance**								
Private	10.8(26)	3.1(5)	2.1(5)	3.1(5)	11.7(28)	4.4(7)	2.91(1.24–6.82)	2.85(1.14–7.16)
Public/not insured	13.6(47)	10.1(16)	3.5(12)	1.3(2)	15.1(52)	10.7(17)	1.48(0.83–2.66)	1.69(0.91–3.15)
**Current Partner**								
Yes	11.1(48)	4.9(13)	2.3(10)	0.4(1)	12.3(53)	4.9(13)	2.74(1.47–5.14)	3.09(1.59–6.03)
No	16.5(26)	10.8(9)	4.5(7)	7.2(6)	17.7(28)	14.5(12)	1.27(0.61–2.66)	1.49(0.68–3.26)
**Cohabitation status (only known for participants reporting a current partner)**								
Yes	6.9 (16)	6.1 (6)	2.2 (5)	0	7.7 (18)	6.1 (6)	1.28 (0.50–3.34)	2.07 (0.77–5.57)
No	16.9 (32)	4.2 (7)	2.7 (5)	0.6 (1)	18.5 (35)	4.2 (7)	5.20 (2.24–12.01)	4.31 (0.53–4.02)
**Depressive Symptoms (CES-D)**								
Low (<10)	5.8(17)	4.1(8)	1.4(4)	1.5(3)	7.1(21)	5.1(10)	1.42(0.66–3.09)	1.73(0.5–3.98)
Medium (10–14)	11.9(16)	7.7(6)	3.7(5)	2.6(2)	13.3(18)	7.7(6)	1.85(0.70–4.87)	2.17(0.78–6.08)
High (≥15)	25.0(38)	9.6(7)	5.3(8)	2.7(2)	25.7(39)	11.0(8)	2.80(1.24–3.36)	2.72(1.13–6.55)
**Termination type**								
Medical	13.6(38)	4.8(9)	2.9(8)	1.1(2)	14.7(41)	4.8(9)	3.39(1.60–7.15)	3.21(1.44–7.18)
Surgical	11.2(35)	7.4(12)	2.9(9)	3.1(5)	12.5(39)	9.2(15)	1.40(0.75–2.63)	1.77(0.91–3.45)
**Alcohol drinks/week**								
<4	11.1(48)	3.6(7)	2.6(11)	2.5(5)	12.0(52)	5.1(10)	2.56(1.27–5.15)	3.08(1.45–6.53)
4.1–14	14.7(11)	9.0(7)	5.4(4)	1.3(1)	17.3(13)	9.0(7)	2.13(0.80–5.67)	4.02(1.35–11.97)
>14	19.1(8)	13.5(7)	4.9(2)	1.9(1)	21.4(9)	13.5(7)	1.75(0.59–5.19)	1.58(0.51–4.90)
**Binge Drinking**								
Yes	12.9(30)	8.7(16)	3.9(9)	2.7(5)	15.1(35)	9.2(17)	1.75(0.94–3.23)	1.87(0.97–3.60)
No	12.2(42)	3.2(5)	2.3(8)	1.3(2)	12.8(44)	4.5(7)	3.11(1.37–7.07)	4.15(1.73–9.96)

^a^ Denominators are the actual number of women within the subcategory who replied to each abuse question

^R^ Referent group is women without children in the month prior to pregnancy.

In addition, we found that women with a current partner had three times the odds of abuse if they had children in the home than if they did not, while no increase was found among women without a current partner (violence perpetrated by a former partner). We previously reported on the high risk to women of physical and sexual abuse by former partners among women in this sample (7.3%) [17]. In this analysis we found that women not living with their current partner had a non-significant but greater increased odds for abuse with children in the home (4.31; 0.53–4.02) than women living with their current partner (2.07; 0.77–5.57).

We explored the possibility of interaction between relationship status (i.e., current partner, former partner, unknown), cohabitation (for women with a current partner only), and risk of abuse by presence of children <18 in the household. We conducted a logistic regression analysis for the subsample of women with a current partner where physical/sexual abuse was the outcome, and controlled for participant age (3 categories) and presence of children in the household (yes/no). Women living with children and in a current relationship but not cohabitating with that person were at nearly 11 times the risk of physical or sexual abuse perpetrated by a former partner (Table 3). This risk estimate was not altered when controlling for length of the current relationship.

**Table 3:** Numbers and age-adjusted odds ratios of physical/sexual IPV by partner status and the interaction variables of cohabitation status and children less than 18 years old living in the home, among participants seeking induced abortion at a women’s health clinic, November 2007 –July 2008.

Cohabitation/child status		IPV by a former or current partner			IPV by a current partner			IPV by a former partner		
	N	IPV + (n)	IPV- (n)	OR (95% CI)	IPV + (n)	IPV- (n)	OR (95% CI)	IPV + (n)	IPV- (n)	OR (95% CI)
**Cohabitation w/ children**	233	18	215	2.29 (0.90–5.82)	11	222	1.73 (0.55–5.38)	8	224	2.39 (0.60–9.5)
**Cohabitation no children**	98	6	92	1.61 (0.52–4.99)	3	95	N/A	3	95	N/A
**Non-cohabiting, w/ children**	189	35	154	6.10 (2.51–14.83)	8	180	1.56 (0.47–5.28)	27	162	10.9 (3.07–38.4)
**Non-cohabiting, no children**	167	7	160	1.00	5	162	1.00	3	164	1.00

## Discussion

Nearly one of every 14 children reported by participants in this study lived in a home in which the woman reported IPV. Furthermore, homes with IPV were associated with younger children (0 to four years of age) when compared with homes in which no IPV was reported. Given these high rates, healthcare visits for family planning, including contraception and pregnancy termination, may provide an important opportunity to intervene on family violence.

Studies from the US, including both urban and rural populations, as well from countries including Haiti, Nepal, India, Turkey and Spain have found that presence of children in the home is associated with increased prevalence of IPV [12, 18–23]. The highest odds of abuse was among the subgroup of women living with children in the home but not cohabitating with their current partner; such abuse was largely perpetrated by a former partner. This group of women seeking elective pregnancy termination had ten times the odds of abuse compared to similar women who were not living with children in the home. We could find no published studies that examined these characteristics, although previous literature has found a strong relationship between abuse by a former partner and homicide [24] as well as IPV leading to injury [25]. One study found that more than half of women killed during pregnancy or the post-partum period were killed by a former partner [24]. For violent relationships in which the partners share a child, the presence of the child may necessitate ongoing partner contact, which may increase exposure to abuse. Other research has indicated that the risk for IPV occurring with children in the home is particularly high when the male partner is not the biological father of the children [25–27]; however, we did not have information about the type of parental relationship to compare to these findings.

We found that women who opted for a medical termination had an increased prevalence of abuse when children were in the home, but no increase among women who opted for surgical termination. Medical termination is required occur early in the pregnancy, as current evidence shows that it is effective through the 9^th^ week of pregnancy [28]. This may indicate that victims of IPV who have children in the home are more decisive or proactive in wanting to end their pregnancy, perhaps because they want to hide the pregnancy from their abusive partner. A meta-analysis of women seeking pregnancy termination found that IPV was associated with non-disclosure to the partner, which is consistent with our interpretation [7]. Although we did not collect data to substantiate this finding, it generates the hypothesis that women who have children and who experience IPV are reluctant to bring more children into the violent situation.

Women who drank less, measured both as drinks per week and binge drinking, and who lived with children had an elevated odds for IPV than women not living with children who drank similar amounts. Women who drank more than 14 drinks per week or who reported binge drinking did not have an elevated odds for abuse based on the presence of children. Although alcohol use has been consistently tied to abuse as both a risk factor and a consequence [29], women with children who are victims of abuse may drink less due to their caregiver role and potentially to be better able to protect both their children and themselves from the violence.

Our study did not measure violence reported by the father, violence against the children, or whether or not the children witnessed the violence. Regardless, the violence experienced by the female head of household will likely have deleterious effects on the children. Few studies have examined the influence of violence victimization on the caregiving role, although existing research theorizes that violence victimization by the mother can negatively influence attachment [30] and that mothers experiencing IPV have needs for support that are not currently being met [31]. The increased prevalence of IPV in homes with children, especially young children, suggest this as an important avenue for future research. Similarly, IPV interventions have focused almost exclusively on the victims or perpetrators, and rarely on the family as a whole. Interventions that incorporate family as well as individual priorities are likely to be most successful and are badly needed.

This study has several limitations. This study was cross sectional so directionality cannot be determined. The sample of women included only the high-risk population of women seeking elective termination of their pregnancies and thus may not generalize to a broader population. We did not ask women about their relationship to the child in the home, nor did we ask about the perpetrators’ relationship to the child. Our survey did not ask specifically about children’s exposure to the violence. We report physical and sexual violence only; battering and emotional abuse could have equally deleterious consequences. Data collection focused on women’s experiences as the target of violence; no questions about the context of the abuse or possibility of bi-directional violence were sought. Finally, with nearly 1,000 participants, this is one of the largest surveys of its kind; however, after stratification by selected covariables, the numbers of subjects were sometimes small.

## Conclusion

This study found that the patients seeking elective pregnancy termination who have children in the home may be at elevated risk for partner abuse, by either current or former partners. An increasing number of healthcare practices are implementing elements of trauma informed care, which helps identify the impact of trauma on health and healthcare delivery [32]. Awareness of family violence can help practitioners better understand the circumstances of their patients, and the implementation of trauma informed care into healthcare practices can help providers tailor care and ensure that appropriate referrals are made.
